# Anti-Inflammatory Effect and Toxicological Profile of Pulp Residue from the *Caryocar Brasiliense*, a Sustainable Raw Material

**DOI:** 10.3390/gels9030234

**Published:** 2023-03-16

**Authors:** Julia Amanda Rodrigues Fracasso, Mariana Bittencourt Ibe, Luísa Taynara Silvério da Costa, Lucas Pires Guarnier, Amanda Martins Viel, Gustavo Reis de Brito, Mariana Conti Parron, Anderson Espírito do Santo Pereira, Giovana Sant’Ana Pegorin Brasil, Valdecir Farias Ximenes, Leonardo Fernandes Fraceto, Cassia Roberta Malacrida Mayer, João Tadeu Ribeiro-Paes, Fernando Yutaka de Ferreira, Natália Alves Zoppe, Lucinéia dos Santos

**Affiliations:** 1 School of Dentistry, São Paulo State University (UNESP), Araçatuba 16015-050, Brazil; 2 Department of Biotechnology, School of Sciences and Languages, São Paulo State University (UNESP), Assis 19806-900, Brazil; 3 Department of Biotechnology and Bioprocesses Engineering, School of Pharmaceutical Sciences, São Paulo State University (UNESP), Araraquara 14800-903, Brazil; 4 Department of Genetics, Ribeirão Preto Medical School, University of São Paulo, Ribeirão Preto 14048-900, Brazil; 5 Nossa Senhora do Patrocínio Faculty Center—CEUNSP, Itu 13300-200, Brazil; 6 Department of Biology, School of Sciences and Languages, São Paulo State University (UNESP), Assis 19806-900, Brazil; 7 Institute of Science and Technology of Sorocaba, São Paulo State University (UNESP), Sorocaba 18087-180, Brazil; 8 Department of Biochemistry and Chemical Technology, Institute of Chemistry, São Paulo State University (UNESP), Araraquara 14800-060, Brazil; 9 Department of Chemistry, Faculty of Sciences, São Paulo State University (UNESP), Bauru 17033-360, Brazil

**Keywords:** Cerrado, inflammation, nanotechnology, pequi, phytotherapic

## Abstract

*Caryocar brasiliense* Cambess is a plant species typical of the Cerrado, a Brazilian biome. The fruit of this species is popularly known as pequi, and its oil is used in traditional medicine. However, an important factor hindering the use of pequi oil is its low yield when extracted from the pulp of this fruit. Therefore, in this study, with aim of developing a new herbal medicine, we an-alyzed the toxicity and anti-inflammatory activity of an extract of pequi pulp residue (EPPR), fol-lowing the mechanical extraction of the oil from its pulp. For this purpose, EPPR was prepared and encapsulated in chitosan. The nanoparticles were analyzed, and the cytotoxicity of the encapsu-lated EPPR was evaluated in vitro. After confirming the cytotoxicity of the encapsulated EPPR, the following evaluations were performed with non-encapsulated EPPR: in vitro anti-inflammatory activity, quantification of cytokines, and acute toxicity in vivo. Once the anti-inflammatory activity and absence of toxicity of EPPR were verified, a gel formulation of EPPR was developed for topical use and analyzed for its in vivo anti-inflammatory potential, ocular toxicity, and previous stability assessment. EPPR and the gel containing EPPR showed effective anti-inflammatory activity and lack of toxicity. The formulation was stable. Thus, a new herbal medicine with anti-inflammatory activity can be developed from discarded pequi residue.

## 1. Introduction

The skin is the largest organ of the human body, and it acts as a physical barrier against the external environment. The protective function of the skin and its annexes may be damaged by aggressive factors, which may lead to various types of injuries, such as physical injuries caused by cuts and sunburn, chemical burns from organic solvents, or viral or fungal infections [1].

In response to these different types of traumas, an inflammatory process is induced in the skin as a defense mechanism to repair damaged tissues [2]. This process is characterized by tissue and functional changes such as vasodilation, increased permeability, and recruitment and activation of leukocytes [3]. These changes are clinically manifested in five main signs that characterize an inflammatory process: heat, pain, redness, edema, and loss of function. When these signals are expressed in an exaggerated manner, the use of anti-inflammatory drugs is necessary [2,3].

In clinical medicine, non-steroidal anti-inflammatory drugs (NSAIDs) are the first choice of treatment for reducing the exaggerated and inappropriate inflammatory response, followed by glucocorticoid anti-inflammatory drugs. However, NSAIDs and glucocorticoids are not effective in many chronic inflammatory processes, and anti-inflammatory drugs such as ketoprofen and ibuprofen generally generate many side effects. Thus, the search for new options in traditional medicine is important for the treatment of inflammation. In particular, medicinal plants can be used as a source of new active ingredients for the development of anti-inflammatory drugs [3].

A striking example of such a medicinal plant is the pequi tree, *Caryocar brasiliense* Cambess. It is the main tree of the Cerrado, the second-largest Brazilian biome. The economic and cultural importance of the pequi fruit for the Cerrado population is well known, especially for those who work in family farming [4]. The pequi tree is the subject of industrial agriculture in Brazil, and the fruit of the pequi is extensively used in food and oil production; therefore, the raw materials are widely available [5].

Additionally, in traditional medicine, pequi pulp oil from the pequi fruit is used in the treatment of various conditions resulting from the inflammatory response [6]. The therapeutic applications of pequi oil are based on the chemical composition of the pequi pulp and almond [7]. The phenolic compounds present in the pequi fruit, mainly flavonoids, exhibit the antioxidant and anti-inflammatory properties observed for its oil, and preclinical studies have confirmed that pequi oil shows anti-inflammatory activity [8,9].

However, an important factor that hinders the use of pequi oil as an anti-inflammatory drug is the low yield of oil extracted from the pulp of this fruit [10]. Nevertheless, pequi pulp residue, a solid residue which results from the oil extraction process through pressing and is usually discarded, contains secondary metabolites that may have anti-inflammatory potential. Spectrophotometric analysis showed the presence of a high concentration of total phenolic compounds in a hydroethanolic extract prepared from this residue [11].

New technologies for drug delivery systems based on plant extracts have been established, including polymeric nanoparticles, which have been widely explored in the development of formulations for topical use. The advantages of nanocarriers compared to conventional topical preparations have been proven; these advantages include improved solubility, pharmacological activity, skin absorption, and formulation stability, in addition to decreased toxicity [12,13,14].

Based on these considerations, with the aim of developing a new herbal medicine which presents good efficacy and low adverse effects, EPPR was initially developed. EPPR was encapsuled in chitosan (CTS), and the cytotoxicity of encapsulated EPPR was evaluated. Because of the cytotoxicity of encapsulated EPPR, analyses of its anti-inflammatory and toxicological potential were performed using non-encapsulated EPPR as well as a gel containing EPPR, which did not present cytotoxicity in a previous study [11].

## 2. Results and Discussion

### 2.1. Flavonoid Content of EPPR

After oil extraction from pequi pulp, the residue generated is normally discarded. The composition of the flavonoids remaining in the EPPR was verified (Table 1).

Barreto et al. [15] showed that the content of flavonoids in the hydroalcoholic extract of pequi pulp is 7.41 mg catechin equivalent (CE)/g. Thus, this result demonstrated the high added value for this residue because the flavonoids, which are phenolic compounds, represent a main group of substances with pharmacological activities in plants [16]. Similarly, in our previous study, Frasao et al. [17] evaluated the ethanolic extract of pequi residue (epicarp and external mesocarp) and verified a low flavonoid content of only 1.64 mg of quercetin equivalent per gram dry weight of the sample (mg QE/g). In our case, the EPPR had a higher value, showing remarkable results.

Other phenolic compounds of EPPR were identified by Pegorin Brasil et al. [11] via ultra-high-performance liquid chromatography coupled with quadrupole-time-of-flight mass spectrometry (UHPLC-ESI-QTOF-MS). These compounds included chlorogenic acid, *p*-coumaric acid, coumaroylquinic acid, caffeic acid glycoside, and sugars (sucrose, methyl-rhamnose-glucose, and rhamnose-galactose-fucose).

Additionally, a comparison was made between the total phenolic compounds in EPPR (21.56 mg gallic acid equivalent (GAE)/g) determined using spectrophotometry by Pegorin Brasil et al. [11] and data already published in the literature for extracts obtained from the pequi residue. The result of the comparison indicated that the content of the total phenolic compounds in EPPR is higher than that reported by Frasão et al. [17], who showed that the ethanolic extract of epicarp and external mesocarp of pequi presented 3.77 mg GAE/g, and by Roesler et al. [18], who revealed total phenolic values of 15.03 mg GAE/g for the ethanolic residue of pequi pulp/seed.

The difference in the content of phenolic compounds and flavonoids in the pequi residue can probably be attributed to the processes used to remove the oil from the pulp, the solvents used in the preparation of the extracts, and the use of different parts of the fruit in the extraction process.

Furthermore, the total phenolic compound content of different extracts of pequi pulp was lower than that reported by Pegorin Brasil et al. [11], Magalhães et al. [19] (1.09 mg GAE/g, aqueous extract), Nascimento-Silva et al. [20] (0.78 mg GAE/g, hydroethanolic extract), and Ribeiro et al. [21] (1.78–3.34 mg GAE/g, ethanolic extract). These results suggest that after the extraction of the oil from the pequi pulp, which corresponds to approximately 35% of the weight of its pulp, the phenolic compounds present in the pulp residue appear proportionately more concentrated because they are water-soluble [22].

These results revealed the high added value of the pequi pulp residue, because the phenolic compounds were preserved even after oil processing.

### 2.2. Characterization of CTS Nanoparticles Containing EPPR

**Figure 1:**
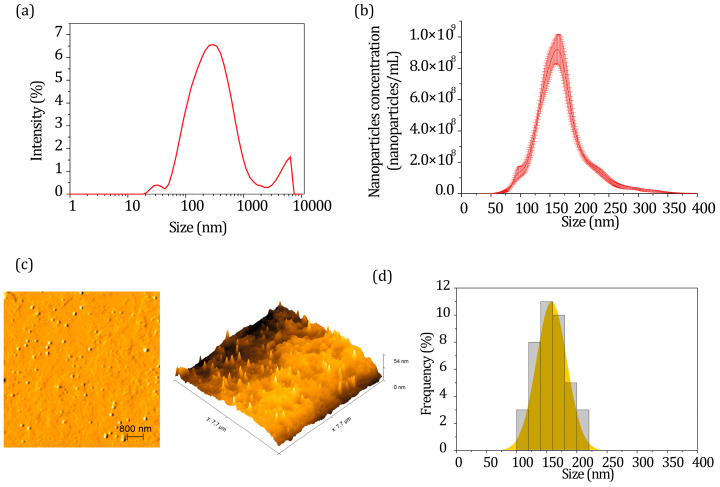
CTS/TPP-EPPR nanoparticle characterization: (**a**) size distribution by DLS (intensity), (**b**) size distribution and nanoparticles concentration by NTA, (**c**) AFM images (topography image, 3D image, and histogram graphic, from right to left, respectively) and (**d**) size distribution by frequency.

CTS/tripolyphosphate (TPP)-hydroethanolic EPPR was characterized using three different techniques: dynamic light scattering (DLS), nano tracking analysis (NTA), and atomic force microscopy (AFM) (Figure 1).

According to the DLS results (Figure 1a), the hydrodynamic size of the nanoparticles was 189 ± 8 nm; the polydispersity index (PDI) was 0.45 ± 0.03; and the zeta potential was +26 ± 1 mV. According to NTA analysis (Figure 1b), the nanoparticles showed a hydrodynamic size of 171 ± 3.6 nm and a concentration of 2 × 10^10^ ± 4 × 10^9^ nanoparticles/mL, which is consistent with the DLS results. AFM images (Figure 1c) showed nanoparticles with a spherical morphology and a mean size of 158 ± 25 nm.

CTS nanoparticles are used for the encapsulation of bioactive compounds [23]. CTS polymers offer great advantages, such as biocompatibility, biodegradability, and nontoxicity for nanoencapsulation [23]. Furthermore, CTS has mucoadhesive properties that enhance the paracellular transport of bioactive compounds, opening the space between epithelial cells and improving bio-compound delivery and bioavailability [24].

Nanoparticle formation occurs via the ionic-gelation method, an intramolecular interaction between TPP and the positive charge of amino groups from CTS. This type of interaction results in pH-stimuli-responsive nanocarrier systems, followed by a sustained release of active compounds [25].

The results obtained in this study showed nanoparticles with the same characteristics of size, surface charge, and morphology as that described in the literature for bio-compounds such as essential oils or plant extracts [26]. According to Mondéjar-López et al. [27], CTS nanoparticles loaded with garlic essential oil have sizes of approximately 172–352 nm. The authors additionally obtained nanoparticles with a positive surface charge (+19 to +48 mV). Mahmoudi et al. [26] used CTS nanoparticles to encapsulate *Physalis alkekengi* L. extract. The nanoparticles had a size of approximately 167 nm and a spherical morphology. In this study, the nanoparticles had a surface charge near neutral (7.69), which the authors attributed to the presence of polyphenol from the extract on the nanoparticle surface.

Owing to the amino groups in the CTS structure, CTS nanoparticles present a positive charge, which can increase or decrease according to the pH. The nanoparticle surface is an important characteristic of nanoformulation stability. A high zeta potential value increases repulsion between the nanoparticles, preventing aggregation and precipitation over time [28].

The nanoparticles prepared in this study showed good colloidal characteristics, and they could be used as a nanocarrier system for EPPR.

### 2.3. Determination of Toxicity of the Encapsulated EPPR by the 3-(4,5-dimethylthiazol-2-yl)-2,5-diphenyltetrazolium Bromide (MTT) Assay

**Figure 2:**
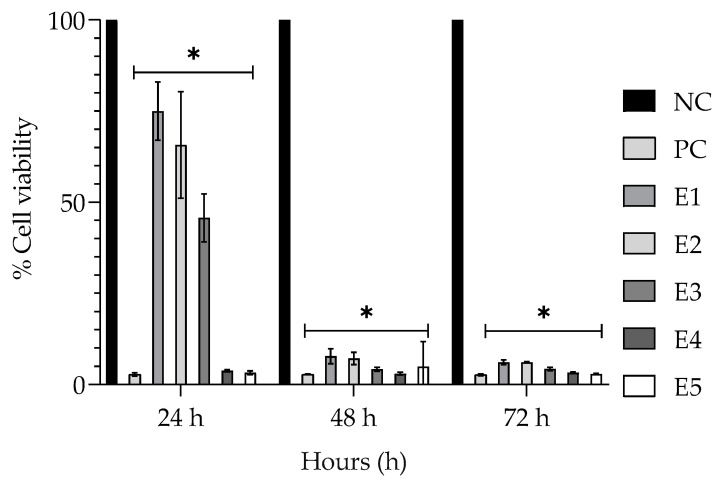
Mean ± SD of the percentage cell viability in the groups treated with the negative control (NC; physiologic solution 0.9%), positive control (PC; 2% Tween 80%), and encapsulated EPPR (E1—31.25 μg/mL, E2—62.5 μg/mL, E3—125 μg/mL, E4—250 μg/mL, and E5—500 μg/mL), according to the MTT method. One-way ANOVA followed by Tukey’s post hoc test. The asterisk (*) indicates a significant difference (*p* < 0.05) between groups.

The encapsulated EPPR, at all concentrations used in this assay, produced a significantly lower cell viability than the negative control (NC) after 24, 48, and 72 h (Figure 2).

Analysis of the cellular cytotoxicity of a plant extract by measuring its viability guarantees the safety of the final product [11]. In the MTT assay, cell viability was evaluated on the basis of the metabolic activity of the mitochondria. This is because microsomal enzymes can reduce MTT, break down its substrate, and transform it into insoluble blue-violet formazan crystals. The color intensity of formazan crystals measured by spectrophotometry is proportional to the cell viability [21]. Mouse fibroblasts (NIH/3T3) were used for investigating the cytotoxicity of encapsulated EPPR because these cell lines are considered suitable in vitro models for investigating the toxicity potential of substances or products for cosmetic purposes [11].

**Figure 3:**
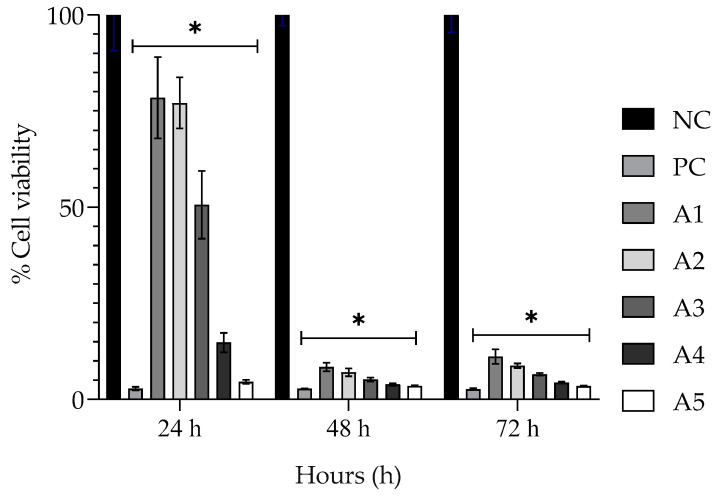
Mean ± SD of the percentage cell viability of the negative control (NC; physiologic solution 0.9%), positive control (PC; 2% Tween 80%), and acetic acid evaluated at five different concentrations (A1—0.015%, A2—0.030%, A3—0.060%, A4—0.120%, and A5—0.240%) by the MTT method. One-way ANOVA followed by Tukey’s post hoc test. The asterisk (*) indicates a significant difference (*p* < 0.05) between groups.

The results of the MTT test showed that the encapsulated EPPR was cytotoxic to fibroblast cells (Figure 3), even at the lowest analyzed concentrations, and the addition of this extract in a formulation proved to be unfeasible. Consistent with the results obtained in this study, Kaisar et al. [29] found that nanoformulations with CTS at a concentration of 500 μM significantly reduced cell viability.

In contrast, Pegorin Brasil et al. [11] reported that mouse fibroblasts (NIH/3T3) exposed to 15.6 to 250 µg/mL EPPR (non-encapsulated extract) for 24 h showed cell viabilities of 132, 132, 138, 134, and 119%, respectively. The cell viability for only the group treated with the highest concentration was statistically equal to that of NC, whereas the cell viabilities for the other groups were higher. After 48 h, the cell viabilities increased slightly to 129, 134, 140, 138, and 136%, respectively, except for that in the group treated with 15.6 µg/mL. All treatments were statistically superior to the NC. However, at 72 h, the viability reduced, reaching 105, 116, 123, 112, and 102%, respectively. Again, all treatments were statistically superior to the NC. Therefore, EPPR did not demonstrate cytotoxic effects, and the cell viability was not dose-dependent. In another independent experiment, Pegorin Brasil et al. [11], using higher concentrations of EPPR (from 625 to 20,000 µg/mL), obtained an IC50 value >2500 µg/mL, that is, the extract inhibited cell proliferation above this concentration.

To understand the cytotoxic effect of the encapsulated EPPR, acetic acid was analyzed by MTT assay at the same concentrations that were used for EPPR encapsulated with CTS. In this analysis, pure acetic acid showed the same cytotoxicity profile as the encapsulated EPPR (Figure 3).

The results obtained in this study suggest that acetic acid may be responsible for the toxicity of the extract in fibroblast cells. Doughty et al. [30] showed that acetic acid is non-toxic to human fibroblast cultures only at concentrations below 0.0025%. As Pegorin Brasil et al. [11] did not observe cytotoxicity for the non-encapsulated extract (EPPR), all analyses presented below were performed with this extract.

### 2.4. Determination of In Vitro Anti-Inflammatory Activity of Non-Encapsulated Extract (EPPR)

#### 2.4.1. Phagocytosis

**Figure 4:**
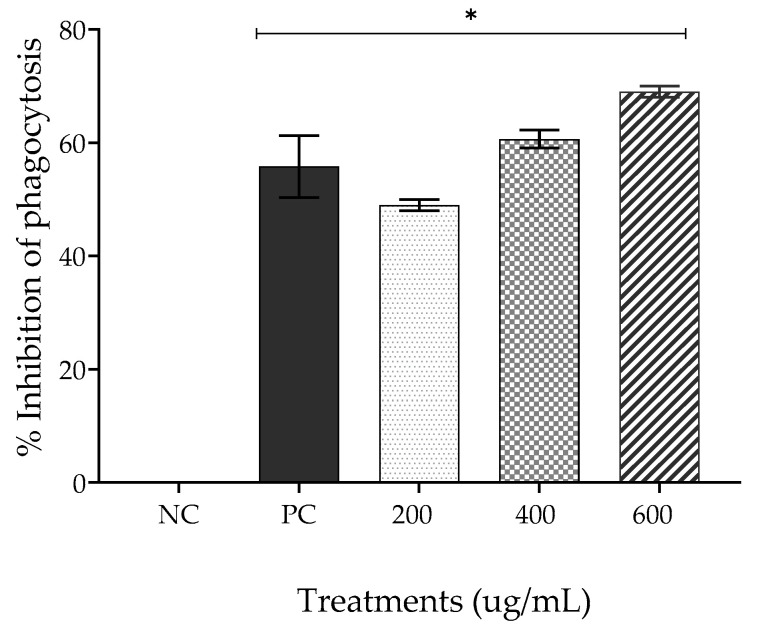
Mean ± SD of the percentage inhibition of phagocytosis for each treatment group: negative control (NC; physiologic solution 0.9%), positive control (PC; 100 μg/mL, dexamethasone), and EPPR (200 μg/mL, 400 μg/mL, and 600 μg/mL). The asterisk (*) indicates a significant difference (*p* < 0.05) compared with the NC. One-way ANOVA followed by Tukey’s post hoc test.

The effects of all treatments were significantly different from that of the NC, saline (Figure 4). EPPR inhibited phagocytosis by 50.61 ± 0.96% at 200 µg/mL; 60.49 ± 1.53% at 400 µg/mL; and 69.13 ± 1.00% at 600 µg/mL. The positive control (PC), dexamethasone, inhibited phagocytosis by 55.77 ± 5.42%.

Macrophages exert immunomodulatory effects on skin wound repair, which is a critical process for restoring skin integrity [23]. Tissue repair comprises three sequential and overlapping healing phases: inflammation, proliferation, and remodeling [31,32]. The inflammatory phase involves the formation of clots by platelets and recruitment of phagocytes [33]. Thus, an uncontrolled process of phagocytosis, rather than repair of the injured tissue, can promote chronic damage [33].

The results obtained in this study indicate that EPPR inhibited macrophage phagocytosis in a similar manner to the commercial anti-inflammatory drug dexamethasone 100 μg/mL (PC). EPPR promoted greater inhibition of phagocytosis than PC, even at concentrations of 400 and 600 μg/mL. The literature has no reports on the anti-inflammatory activity of pequi plant extracts. Athira and Keerthi [34] observed that *Sigmadocia* extract produced a low level of phagocytosis.

#### 2.4.2. Spreading

Spreading is generally defined as an unsuccessful attempt at phagocytosis. However, as no substance or microorganism is present to be phagocytosed, spreading is the action of a responsive cell with the ability to adhere to the slide and emit microvilli [16].

**Figure 5:**
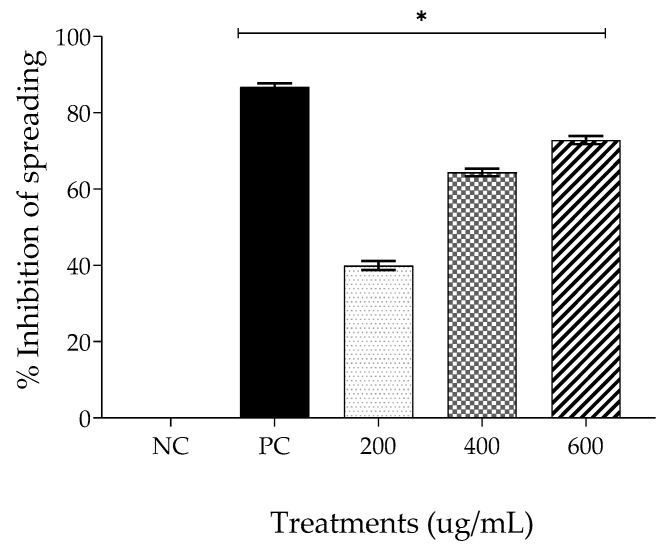
Mean ± SD of inhibition of spreading for each treatment group: negative control (NC; physiologic solution 0.9%), positive control (PC; 100 μg/mL, dexamethasone), and EPPR (200 μg/mL, 400 μg/mL, and 600 μg/mL). The asterisk (*) indicates a significant difference (*p* < 0.05) compared with the NC. One-way ANOVA followed by Tukey’s post hoc test.

All treatments used in this assay produced significantly different effects compared with the NC (*p* < 0.05) (Figure 5). EPPR promoted the inhibition of spreading in the following proportions: 39.95 ± 1.17% at 200 µg/mL; 64.36 ± 0.99% at 400 µg/mL; and 72.84 ± 1.07% at 600 µg/mL. This assay corroborates the results of the phagocytosis assay, demonstrating that EPPR can reduce inflammation symptoms in macrophages. This capacity is similar to that of 100 µg/mL dexamethasone, which reduced spreading by 86.74 ± 0.94%.

#### 2.4.3. Membrane Stabilization

The human red blood cell stabilization method was used to analyze the anti-inflammatory activity of the plant extracts [19]. According to Kumar et al. [35], during the inflammatory response, anti-inflammatory drugs interfere with different biochemical processes, promoting several effects, including the stabilization of lysosome membranes. Thus, because the structure of the erythrocyte membrane is analogous to that of the lysosomal membrane, the ability of a substance to promote its stabilization may be a predictive factor for its anti-inflammatory activity [35].

**Figure 6:**
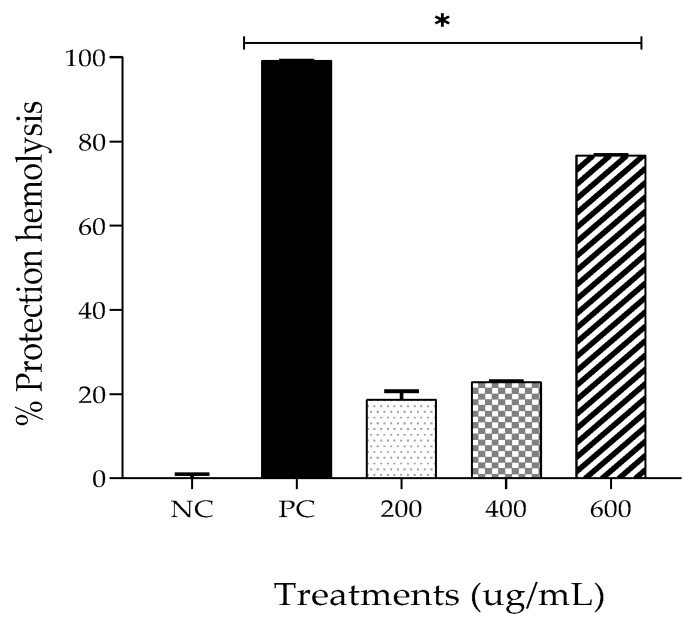
Mean ± SD of the percentage of protection against hemolysis for each treatment group: negative control (NC; physiologic solution 0.9%), positive control (PC; 100 μg/mL, dexamethasone), and EPPR (200 μg/mL, 400 μg/mL, and 600 μg/mL). The asterisk (*) indicates a significant difference (*p* < 0.05) compared with the NC. One-way ANOVA followed by Tukey’s post hoc test.

Figure 6 shows that according to the erythrocyte membrane stability test, all treatments used in this assay produced significantly different effects compared with the NC (*p* < 0.05). EPPR promoted the protection of the red blood cell membrane in the following proportions: 18.71 ± 2.01% at 200 μg/mL; 22.85 ± 0.20% at 400 μg/mL; and 76.67 ± 0.22% at 600 μg/mL. Protection from hemolysis promoted by the PC, dexamethasone, was 99.11 ± 0.32%.

Kumar et al. [35], using the membrane stabilization test, evaluated the anti-inflammatory activity of the extract of leaves of the plant *Skimmia anquetilia*, originally from India; the extract exhibited a protection of 68.40% at a concentration of 400 mg/mL. Dias [36] analyzed the microencapsulated essential oil of *Lippia pedunculosa* and observed 100% inhibition of hemolysis at concentrations of 10, 50, 100, and 250 µg/mL. Sousa et al. [37] evaluated the extract of *Pavonia glazioviana Gürke* (Malvaceae) at 500 and 1000 µg/mL and reported a protective effect of less than 60%.

Other analyses have shown that pequi oil protects against inflammation [9]; however, no information is available regarding the residue from pequi oil extraction. The three in vitro tests performed in this study show that EPPR exhibits effective anti-inflammatory activity, and that the best results were obtained at a concentration of 600 µg/mL.

### 2.5. Quantification of the Levels of Cytokines IL-6 and IL-10 Induced by the Non-Encapsulated Extract (EPPR)

Studies have established that lipopolysaccharides (LPS) induce the secretion of pro-inflammatory cytokines such as TNF-α and interleukins [38]. Cytokines are signaling polypeptides used in cell communication by the immune system during the inflammatory response; they act on virtually all cell types and in mRNA synthesis [38]. Interleukin-6 (IL-6) is the most important mediator of the acute inflammatory response and is the main procoagulant cytokine [3,38]. IL-10 has anti-inflammatory and suppressive effects on most hematopoietic cells and indirectly suppresses the production of other cytokines. However, IL-10 has also been shown to produce stimulatory effects on CD8+ effector T cells, increasing their cytotoxic and proliferative capacities [3]. Thus, IL-10 is increased in certain pathologies, such as HIV (human immunodeficiency virus) and Burkitt’s lymphoma [39]. In addition, dysregulation of IL-10 is associated with enhanced hyperinflammation in response to infection, as well as an increased risk for the development of many autoimmune diseases. Thus, an understanding of IL-10 participation in the progression and resolution of certain inflammatory-response-related diseases is critical [40].

**Figure 7:**
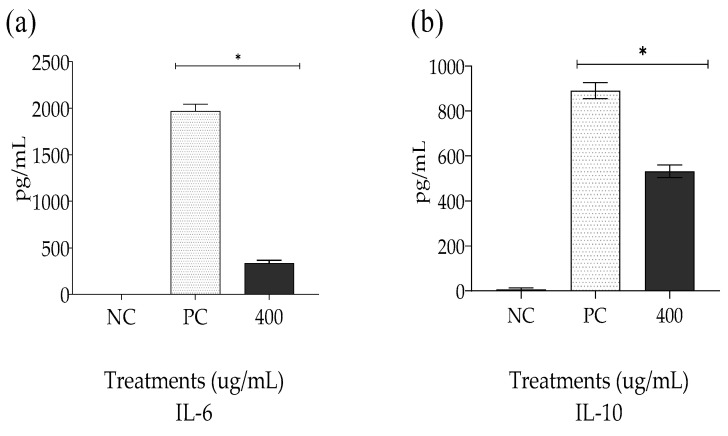
Mean ± SD of the levels of the interleukins IL-6 (**a**) and IL-10 (**b**) (pg/mL) for each treatment group: negative control (NC; untreated cells), positive control (PC; LPS), and EPPR (400 µg/mL). The asterisk (*) indicates a significant difference (*p* < 0.05) compared with the NC. One-way ANOVA followed by Tukey’s post hoc test.

The results of the present analysis, as expected, showed that the group treated with LPS as the PC showed a significant increase (*p* < 0.05) in the production of IL-6 (Figure 7a) and IL-10 (Figure 7b) when compared with the NC group of untreated cells. Conversely, treatment with EPPR (400 µg/mL) significantly (*p* < 0.05) prevented LPS-induced increase in interleukin concentrations.

To date, no study has reported on extracts obtained from pequi residue. However, Torres et al. [41] reported that pequi oil reduced IL-6 expression. The EPPR-induced decrease in the levels of the pro-inflammatory mediator IL-6 complements the results of the in vitro tests on the anti-inflammatory effect of EPPR.

### 2.6. Acute Toxicity In Vivo of the Non-Encapsulated Extract (EPPR)

EPPR administered in a single dose of 2000 mg/kg did not promote the death of animals. The results obtained during 14 days of analysis of the manifestation of toxic signs showed that among all the parameters analyzed concerning motor control and consciousness, only slight signs of CNS hyperexcitability and hypnosis were manifested by the animals in the first 4 h after EPPR administration (Table 2). These aspects were no longer observed after this period.

Considering that on the scale, acute toxicity “0” indicated absence of the effect analyzed and “4” indicated the maximum effect, we verified that EPPR promoted subtle signs of toxicity in the first 4 h of evaluation. Thus, in the acute toxicity test in animals, EPPR did not cause mortality, morbidity, unusual behavior, or severe and permanent adverse clinical signs. In addition, EPPR did not kill the animals at a dose of 2000 mg/kg after a single oral administration. Thus, this extract can be classified as a Class 4 drug, according to the acute toxicity classification criteria for chemicals [17,19,42].

In addition, on the 14th day of EPPR administration, the EPPR-treated animals exhibited a significant increase in weight compared with the control animals (*p* < 0.05) (Table 3).

According to Di Santo et al. [43], when the plant extract stimulates appetite and, thus, increases the body weight of animals, it tends to behave as a non-toxic substance, as it does not harm the physiological functions of the organism and allows the regular execution of metabolism.

### 2.7. Determination of the In Vivo Anti-Inflammatory Activity of the Gel Containing the Non-Encapsulated Extract (EPPR gel) on Carrageenan-Induced Paw Edema

The paw edema test in rodents has been well established for the evaluation of new drugs. In this test, the development of carrageenan-induced paw edema occurs in two phases. In the initial phase (0–1 h), serotonin, histamine, bradykinin, and substance P are released. The second phase (after 1 h) involves invasion of mainly neutrophils at the site of inflammation and production of large amounts of pro-inflammatory mediators, such as PGE2, and various cytokines, such as IL-6 and IL-10 [44].

EPPR gel (5 mg/g) and dexamethasone gel (1 mg/g, PC) reduced carrageenan-induced edema, which was determined by measuring the paw volume (mL) of the animals. The reduction of edema was considered an anti-inflammatory activity. Gels containing dexamethasone and EPPR showed a significant (*p* < 0.05) reduction in carrageenan-induced paw edema when compared with the NC. This reduction was observed from the second hour onwards and remained until the sixth hour after the application of carrageenan. More specifically, in the second hour, the NC, PC, and EPPR gels showed, respectively, the following edema values: 0.08 ± 0.005 mL, 0.04 ± 0.002 mL, and 0.04 ± 0.006 mL. In addition, the gels containing EPPR and dexamethasone inhibited edema by 49.95% and 53.33%, respectively (Table 4).

**Figure 8:**
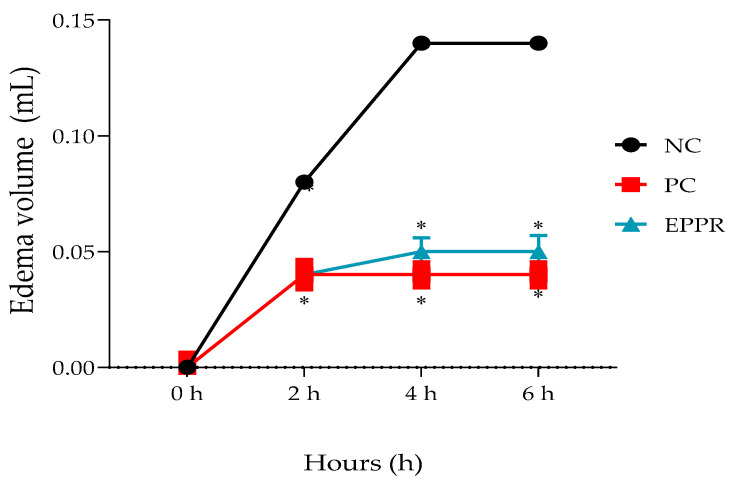
Percentage of edema inhibition in mL (n = 8/group) by the negative control (NC; base gel), positive control (PC; dexamethasone gel 1 mg/g), and EPPR (EPPR gel, 5 mg/g). One-way ANOVA followed by the Tukey–Kramer multiple comparison tests showed * *p* ˂ 0.005 compared with the NC group.

Figure 8 shows that from the fourth to the sixth hour of carrageenan administration, dexamethasone gel (PC) and EPPR gel exhibited anti-inflammatory activity. The edema values with these treatments differed significantly from those with the base gel (NC) treatment (*p* ˂ 0.05) and remained constant. The NC, PC, and EPPR gel treatments resulted in the following edema volumes per hour: fourth hour (0.14 ± 0.006, 0.04 ± 0.004, and 0.05 ± 0.005, respectively) and sixth hour (0.14 ± 0.01, 0.04 ± 0.01, and 0.05 ± 0.01, respectively). The PC and EPPR gel treatments, respectively, inhibited edema by similar extents at the fourth and sixth hours: 70.27% and 67.79%.

Anti-inflammatory in vivo tests using formulations containing pequi oil are well established in the literature. However, nothing is known regarding the anti-inflammatory effects of topical products containing EPPR. Diniz [45] found a significant reduction in paw edema 1 h after administration of a microemulsion containing pequi oil. Bezerra [6] verified the anti-inflammatory effect of an emulsion with pequi oil and observed that the application of the emulsion in the paws of mice significantly reduced the release of myeloperoxidase (MPO) in neutrophils by 64.8%. Santos et al. [46] reported that the application of pequi oil (700 and 1000 mg/kg) resulted in a significant decrease in paw edema (64% and 79%), reaching its maximum inhibition peak. Thus, this study verified that the gel containing the pequi residue showed equal activity to that of its oil form. These results are probably owing to the large number of phenolic compounds present in the EPPR, particularly catechin.

### 2.8. In Vitro Ocular Irritability Test of the Gel Containing the Non-Encapsulated Extract (EPPR gel) in the Chorioallantoic Membrane of Chicken Eggs

The final classification of the degree of irritation of the gels was based on the average of the sum of the values obtained by the three samples per group. The results of the in vitro irritability test (Table 5) showed a score of zero (non-irritant) for saline solution (SS) and a score of 21 (severe irritant) for NaOH (PC). All other treatments were classified as non-irritants or mild irritants (MI), suggesting that they can be safely applied to the skin. Mansur et al. [47] reported that antioxidant extracts did not cause irritation, corroborating our findings.

### 2.9. Preliminary Stability Evaluation of the Gel Containing the Non-Encapsulated Extract (EPPR Gel)

Finally, with considerations for the possibility of developing an herbal medicine according to the demands of the international consumer market, the stability of the formulations was evaluated. In the centrifugation test, the base gel and EPPR gel did not show any changes. The thermal stress test verified that none of the samples of the gel presented separation into phases upon reaching a temperature of 80 °C. The pH values of the base gel (the gel without the extract) and the EPPR gel did not present any statistically significant differences. The EPPR gel showed a mean pH of 5.91 ± 0.05, and the base gel showed a mean pH of 5.54 ± 0.06. The values were within the threshold range of compatibility with the physiological pH of the skin, which ranges from 5.5 to 7.3.

## 3. Conclusions

In conclusion, the chromatographic quantification of EPPR showed a significant presence of flavonoids, suggesting a relationship between the pharmacological activity of this extract and its phytochemical composition. The nanoparticles prepared in this study with CTS showed good colloidal characteristics and could be used as a nanocarrier system for EPPR. However, in the MTT assay, the encapsulated EPPR showed cytotoxicity due to acetic acid. EPPR, a non-encapsulated extract, showed anti-inflammatory activity in vitro. In addition, it significantly reduced the concentration of the inflammatory cytokines IL-6 and IL-10 and showed no toxicity in vivo. Furthermore, in vivo testing of the EPPR gel showed anti-inflammatory activity similar to that of the PC, dexamethasone, an anti-inflammatory agent widely used in clinical medicine. Lastly, the formulation of the EPPR gel showed stability and a lack of toxicity in the ocular test. In conclusion, it is possible to develop a new herbal medicine from pequi residue that is normally discarded for the treatment of inflammatory skin diseases.

## 4. Materials and Methods

### 4.1. Material of Vegetable Origin

**Figure 9:**
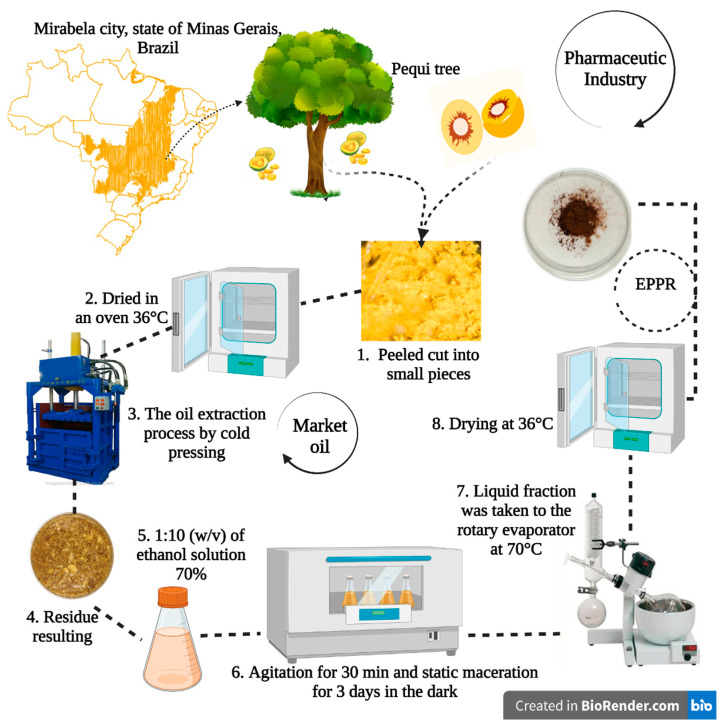
Preparation of the hydroethanolic extract of pequi pulp residue (EPPR).

Pequi fruits were obtained from the city of Mirabela, state of Minas Gerais, in the southeast region of Brazil (latitude: 16°15′46″ S; longitude: 44°09′52″ W, altitude: 800 m). The fruits were first washed and peeled and subsequently packed in plastic bags and stored under refrigeration (−18 °C; Figure 9). The species under study was identified as *Caryocar brasiliense* Cambess in the Herbarium of the Universidade Estadual Paulista (UNESP), where a voucher specimen was deposited under the number 1998. This study was registered in the National System for the Management of Genetic Heritage and Associated Traditional Knowledge (SisGen) under license no. A23C398.

Fruits were washed, peeled, manually cut into small pieces, and subjected to the oil extraction process by pulp cold pressing. The residue resulting from the pulp pressing was dried in an oven (36 °C).

### 4.2. Preparation of the Hydroethanolic *EPPR*

The hydroethanolic EPPR was prepared at a proportion of 1 g of dry pequi pulp residue per 10 mL of ethanol solution (70%, *v*/*v*), as shown in Figure 9. Subsequently, it was subjected to vigorous agitation for 30 min and kept in static maceration for 3 days in the dark. The resulting extract was filtered to obtain a liquid fraction. This fraction was placed in a rotary evaporator (Fisatom^®^, São Paulo, SP, Brazil) under reduced pressure at 70 °C to eliminate the alcohol and placed in an oven (Solab^®^, Piracicaba, SP, Brazil) to dry at 36 °C for the complete elimination of water and to obtain a constant weight of the dried EPPR [48].

### 4.3. Flavonoid *Content* of EPPR

The flavonoid content of EPPR was determined using the method described by Serdar et al. [49]. An analytical curve was constructed using solutions of 10–100 μg/mL quercetin (y = −0.0039 + 0.0082x and R2 = 0.9999). The absorbance of the samples (solubilized in ethanol) was measured at 425 nm using a UV–Vis spectrophotometer (800× XI, Femto^®^, Brazil). The experiments were performed in triplicates. The results were expressed in mg QE/g.

### 4.4. *Encapsulation* of EPPR in CTS via Ionic Gelatinization

CTS used in this study was a commercial product (CAS 9012-76-4, Sigma Aldrich^®^, St. Louis, MO, USA). Encapsulation was performed according to the protocol proposed by Calvo et al. with modifications [50]. The CTS nanoparticles were obtained by gelation of a CTS solution with polyanion sodium tripolyphosphate. For this purpose, initially, CTS was dissolved in aqueous solutions of acetic acid at various concentrations (0.015%, 0.030%, 0.060%, 0.120%, and 0.240%). The concentration of acetic acid was, in all cases, 1.2 times higher than that of CTS. The dry EPPR was incorporated into the QTS solution at the following concentrations: 31.25, 62.5, 125, 250, and 500 μg/mL

### 4.5. Characterization of CTS Nanoparticles Containing EPPR

#### 4.5.1. DLS

The nanoparticles were characterized by DLS to obtain their hydrodynamic size (nm) and PDI. The analyses were performed using ZetaSizer (model ZS90, Malvern Instruments^®^, Malvern, UK) with a scattering detection angle of 90°. The analysis was performed in triplicates at 25 °C.

#### 4.5.2. Surface Charge

The nanoparticle surface charge (zeta potential) was determined using electrophoresis. Analyses were performed using ZetaSizer equipment (model ZS90, Malvern Instruments^®^, UK). The analysis was performed in triplicates at 25 °C.

#### 4.5.3. NTA

The size distribution and the nanoparticle concentration were determined by NTA (model LM-10, Malvern Instruments^®^, UK). The samples were diluted 100× in ultrapure water, the analyses were performed five times, and 400 nanoparticles were counted for each measurement. The samples were stored at 25 °C.

#### 4.5.4. AFM

Images were obtained using the easyScan 2 basic AFM system (Nanosurf^®^, Liestal, Switzerland). The samples were scanned in contact mode using TapAl-G cantilevers (BudgetSensors^®^, Sofia, Bulgaria). The nanoparticles were diluted 1000× in ultrapure water. The size of the nanoparticles was determined by analyzing the images using the ImageJ^®^ software (Bethesda, MD, USA).

### 4.6. Determination of Toxicity of the Encapsulated EPPR by the MTT Assay

The MTT cytotoxicity assay was performed as described previously by Tsuboy et al. [51]. For this assay, mouse fibroblasts of dermal origin (NIH/3T3, ATCC^®^ CRL-1658™) were inoculated into 96-well microtiter plates and incubated in culture medium for 24 h at 37 °C under 5% carbon dioxide (CO_2_). After reaching approximately 75% confluence (24 h), these cells were exposed to five different concentrations of the encapsulated EPPR (31.25, 62.5, 125, 250, and 500 μg/mL). In addition, to understand the cytotoxicity of the encapsulated EPPR, acetic acid was evaluated at the same proportions used in the encapsulation process (0.015%, 0.030%, 0.060%, 0.120%, and 0.240%). For the NC, the extract was replaced with a physiological solution, and for the PC, it was replaced with 2% (*v*/*v*) Tween 80. The treatment times were 24, 48, and 72 h.

EPPR was not included in this screening because Pegorin Brasil et al. [10] analyzed the same extract and showed that it did not present significant cytotoxicity.

### 4.7. Determination of In Vitro Anti-Inflammatory Activity of the Non-Encapsulated Extract (EPPR)

#### 4.7.1. Treatments

In all anti-inflammatory tests, the samples analyzed were EPPR. The dry EPPR was dissolved in distilled water at the following concentrations: 200, 400, and 600 μg/mL. For the PC, the extract doses were replaced with dexamethasone (100 μg/mL), and the NC was replaced with saline solution. In the hemolysis stabilization test, the saline solution was replaced with a hyposaline solution (0.18%) to induce hemolysis.

#### 4.7.2. Cell Culture

Murine macrophages of the Raw 264.7 (ATCC TIB-71) strain were thawed and cultured in a cell culture flask with Dulbecco’s Modified Eagle Medium (DMEM) Ham’s F-12 culture medium at 37 °C under 5% CO_2_. Cells were grown to 70–80% confluence.

#### 4.7.3. Selection of Macrophages

Cells were harvested using a cell scraper, counted in a Neubauer chamber, and centrifuged at 1500 rpm for 5 min. The supernatant was discarded, and the cells were resuspended in culture medium to reach the desired concentration for each experiment.

#### 4.7.4. Phagocytosis

The method described by Azedo et al. [52] was used in this assay. The prepared slides were examined under an optical microscope at 400× magnification, with a total count of 100 cells. This test was performed in triplicates.

Inhibition of phagocytosis (IP) was calculated using the following formula:

IP (%) = E0 − ET/E0 × 100,(1)

where E0 represents the mean number of cells in the NC group that phagocytosed the zymosan particles, and ET represents the mean number of cells in the treated groups that phagocytosed the zymosan particles.

#### 4.7.5. Macrophage Spreading

The method described by Bastos et al. [53] was used in this study. The prepared slides were examined under an optical microscope at 400× magnification, with a total count of 100 cells. This test was performed in triplicates.

The inhibition of spreading was calculated using the following formula:

Inhibition of spreading (%) = E0 − ET/E0 × 100,(2)

where E0 represents the mean number of spread cells in the NC group, and ET represents the mean number of cells spread in the treated groups.

#### 4.7.6. Membrane Stabilization

The human red blood cell membrane stabilization (HRBC) test was performed according to the method proposed by Ananthi and Chitra [54].

The test reaction was performed by adding 2 mL of hyposaline solution (0.18%), 1 mL of sodium phosphate buffer (0.1 M, pH 7.4), 1 mL of the analyzed samples, and 0.5 mL of HRBC solution. The hemoglobin content in the suspension was estimated using a spectrophotometer at 560 nm.

The percentage of protection can hence be calculated from the equation given below:

Protection (%) = E0 − ET/E0 × 100,(3)

where E0 represents the mean absorbance value of the NC group and ET represents the mean absorbance value in the treated groups.

### 4.8. Quantification of the Levels of the Cytokines IL-6 and IL-10 Induced by the Non-Encapsulated Extract (EPPR)

For cytokine determination, bone-marrow-derived macrophages (BMDMs) from C57BL/6 mice were prepared as previously described by the Organization for Economic Co-operation and Development (OECD) [53]. BMDMs were conditioned in a 96-well bottom plate (Nunc, Thermo Fisher Scientific, Waltham, MA, USA) (2 × 10^5^ cells/well) and stimulated with LPS from *Escherichia coli* (Sigma-Aldrich) at a concentration of 500 mg/mL. After 3 h, the cells were washed with 1× phosphate-buffered saline and treated with EPPR (600 µg/mL) for 18 h. The supernatant was collected, and the cytokines were measured by enzyme-linked immunosorbent assay (ELISA) using a mouse IL-6 and IL-10 kit (R&D Quantikine ELISA) according to the manufacturer’s instructions.

### 4.9. Animals

Twelve-week-old male Swiss mice weighing 31–40 g were housed in polypropylene cages (five animals per box). Food (Nuvilab CR-1 kibble) and water were provided ad libitum. The vivarium was maintained at a controlled temperature (23 ± 2 °C) and humidity (55% ± 10%) with an artificial lighting program that corresponded to 50 lx (lights on at 7:00 a.m. and off at 7:00 p.m.). The study was approved by the local Ethics Committee on the Use of Animals of Universidade Estadual Paulista “Júlio Mesquita Filho”, Assis campus, São Paulo, Brazil (protocol no. 13/2018; approved on 22 March 2018) and conducted according to the Brazilian Legal Framework for the Scientific Use of Animals.

### 4.10. Determination of In Vivo Toxicity of the Non-Encapsulated Extract (EPPR)

This analysis was performed in accordance with the OECD Guideline No. 423 [55]. Male Swiss mice (n = 8) were used for the control and experimental groups. The control group was treated with distilled water (5 mL/kg of body weight). Treatment with dry EPPR dissolved in distilled water was administered by gavage in a single dose to one animal at a time from an initial concentration of 2000 mg/kg of body weight. This dose was determined on the basis of the classification of the OCDE, which defines a harmful substance as being capable of promoting the death of 50% of a test population (LD50) after acute administration of a dose of 200–2000 mg/kg. Physiological aspects were analyzed for 14 days. The analysis scale ranged from 0 to 4, where 0 indicated the absence of the effect analyzed and 4 indicated the total observation. Each group contained eight male animals. The initial and final weights of the animals were measured, and the average values for the group were determined.

### 4.11. Preparation of the Gel Formulation Containing the Non-Encapsulated Extract (EPPR Gel)

To prepare the formulation, 1% (*w*/*w*) carbopol and 0.1% (*w*/*w*) methylparaben, a preservative, were dissolved in water, and the mixture was allowed to rest for 24 h. Subsequently, the mixture was stirred, and dry EPPR was added at a concentration of 5 mg/g. The pH of the solution was adjusted to 5.5.

### 4.12. Determination of the In Vivo Anti-Inflammatory Effect of the Gel Containing the Non-Encapsulated Extract (EPPR Gel) on Carrageenan-Induced Paw Edema

**Figure 10:**
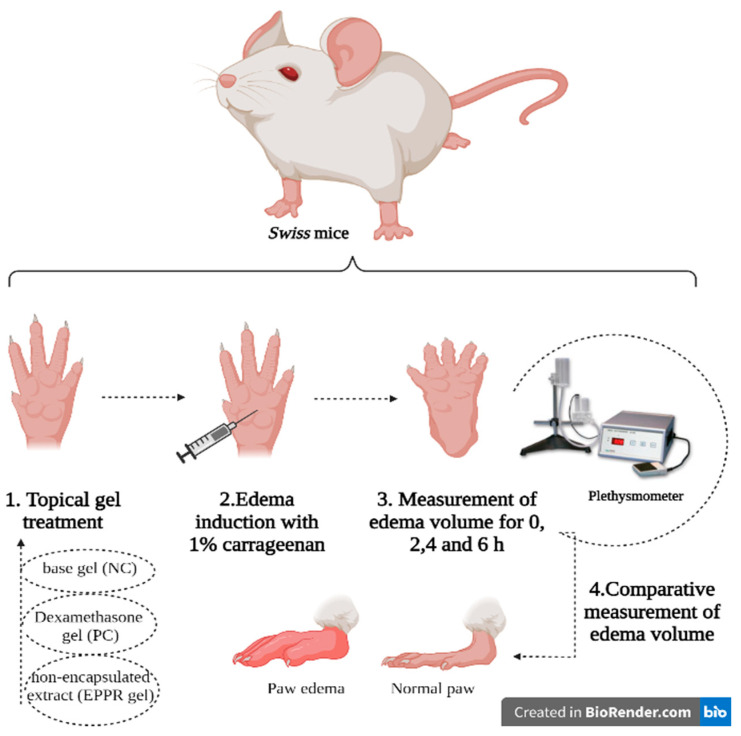
Paw edema model.

According to Winter, Risley, and Nuss [56], male mice (n = 8/group) were subjected to a subplantar injection in the animal’s right hind paw with 0.1 mL of 1% carrageenan. The treatments were EPPR gel (5 mg/g), base gel as the NC, and dexamethasone gel (1 mg/g) as the PC. To assess the acute anti-inflammatory effect, the volume of the right hind paw was measured using a plethysmometer (Ugo Basile^®^, Gemonio, Italy) before the first carrageenan administration and at 0, 2, 4, and 6 h after administration (Figure 10).

The inhibition percentage was calculated using the following formula:

% inhibition = (E0 − ET)/E0 × 100,(4)

where E0 represents the mean volume of paw edema observed in the control group and ET represents the mean volume of paw edema observed in the treated groups.

### 4.13. Ex Vivo Ocular Irritability Test of the Gel Containing the Non-Encapsulated Extract (EPPR Gel) in the Chorioallantoic Membrane of Chicken Eggs (MCA)

This test was performed according to the methodology described in the Journal Officiel de la République Française [57]. Four fertilized eggs from White Leghorn chickens were used per treatment group: NC (saline 0.9%, *w*/*v*), PC (sodium hydroxide 0.1 N), base gel (gel without the extract), and EPPR gel 5 mg/g.

On the tenth day of incubation, the treatments were applied to the MCA, and the presence or absence of irritating effects was observed. After visual analysis, a thiopental solution was injected into the fertilized eggs. The graduation of each phenomenon was determined in 5 min and graduated in numerical values (1, 3, 5, 7, and 9) depending on time (Table 6). Visual analysis of the MCA was performed using a magnifying glass. 

The analyzed samples were classified according to the mean value of the sum of the scores of three independent tests (n = 3), and the degree of irritation was divided into four categories: between 0.0 to 0.99, non-irritating (NI); 1.0 to 4.99, mild irritant (MI); 5.0 to 8.99, moderate irritant (MI); and 9.0 to 21, severe irritant (SI) [44].

### 4.14. Preliminary Stability Evaluation of the Gel Containing the Non-Encapsulated Extract (EPPR Gel)

The formulations with non-encapsulated EPPR were evaluated at a concentration of 5 mg/g. The gel without the extract, the denominated base gel, was used as the negative control. These tests were performed in triplicates.

#### 4.14.1. Accelerated Stability Test or Centrifugation Test

Five grams of each formulation was weighed into centrifuge tubes. The centrifugation test (MEGAFUGE 16R centrifuge, Thermo Scientific^®^, New York, NY, USA) was performed at room temperature with a rotational speed of 210× *g* for 30 min. Subsequently, the formulation was macroscopically analyzed by its appearance.

#### 4.14.2. Thermal Stress Test

Tubes containing 5 g of each formulation were subjected to thermal stress in a thermostat water bath (Solab^®^, São Paulo, SP, Brazil) at temperatures ranging from 40 to 80 °C, with an increase of 10 °C every 30 min up to 80 °C. The formulation was analyzed macroscopically by its appearance after reaching room temperature (25 °C).

#### 4.14.3. pH evaluation 

The pH of the formulation was evaluated weekly for 30 days using a calibrated pH meter (mPA210MS; TECNOPON^®^, Piracicaba, SP, Brazil).

### 4.15. Statistical Analysis

The data are expressed as the mean ± standard deviation. Statistical analyses were performed using the Prism 8. To verify the statistical differences between the groups, a one-way analysis of variance (ANOVA) was performed according to the experimental protocol, followed by Tukey’s multiple comparison test. For all analyses, a *p*-value < 0.05 was considered to indicate statistical significance.

## 5. Patents

The patent for the EPPR gel was granted by Instituto Nacional da Propriedade Industrial (INPI, Brasília, Brazil) on 25 November 2020 with the process number BR 10 2020 024093 5.

## Figures and Tables

**Table 1:** Mean ± standard deviation of the flavonoid content in EPPR. Results expressed in mg of quercetin equivalent per gram dry weight of the extract (mg QE/g).

Sample	mg QE/g
EPPR	5.88 ± 0.15

**Table 2:** Mean of the numerical parameter used for evaluating the toxicological, motor, and consciousness-related effects of a single-dose oral administration of EPPR. The toxicity scale ranges from “0” to “4”.

General Aspects	Evaluation Time after Administration				
	30 min	1 h	2 h	3 h	4 h
CNS Hyperexcitability	0.00	0.11	0.32	0.20	0.00
Hypnosis	0.20	0.15	0.50	0.77	0.40

**Table 3:** Mean ± SD of the body weight (grams) of mice treated with a single dose of EPPR.

Parameter	Control	EPPR
Starting weight	45 ± 10.00	39 ± 15.31
Final weight	51 ± 14.00	57 ± 16.44

**Table 4:** Anti-inflammatory activity of the gels in mean percentage, represented by the ability to inhibit carrageenan-induced paw edema at different time points.

Treatments (Gels)	% Anti-Inflammatory Activity			
	0 h	2 h	4 h	6 h
NC	0%	0%	0%	0%
PC	0%	53.33% *	70.27% *	70.27% *
EPPR	0%	49.95% *	67.79% *	67.79% *

Negative control (NC; base gel), positive control (PC; dexamethasone gel 1 mg/g), and EPPR (EPPR gel, 5 mg/g). n = 8/group. One-way ANOVA followed by the Tukey–Kramer multiple comparison test showed * *p* ˂ 0.005 compared with the NC group.

**Table 5:** Grade average and final ocular irritability rating of saline solution and gels. The numerical grading varies between 1, 3, 5, 7, and 9 depending on the team.

Treatments	Grade Average	Final Rating
SS	0	Non-Irritant
NC	0	Non-Irritant
PC	21	Severe Irritant
EPPR	1	Mild Irritant

Saline solution (SS, 0.9%), negative control (NC; base gel), positive control (PC; dexamethasone gel 1 mg/g), and EPPR (EPPR gel, 5 mg/g).

**Table 6:** Numerical graduation (1, 3, 5, 7, and 9) of the phenomena as a function of the elapsed time (seconds) for their occurrence.

Phenomenon	30 s	30 and 60 s	60 and 300 s
Hyperemia	5	3	1
Bleeding	7	5	3
Coagulation	9	7	5

## Data Availability

The data that support the findings of this research are available from the corresponding author upon reasonable request.

## References

[1] Sabat R., Wolk K., Loyal L., Döcke W.-D., Ghoreschi K. (2019). T Cell Pathology in Skin Inflammation. Semin. Immunopathol..

[2] Santos T.F.B. (2018). Bioprospecting of endophytic filamentous fungi of medicinal species with potential to produce molecules of biotechnological interest. Master’s Dissertation.

[3] Tanaka T., Narazaki M., Kishimoto T. (2014). IL-6 in Inflammation, Immunity, and Disease. Cold Spring Harb. Perspect. Biol..

[4] Santos F.S., Santos R.F., Dias P.P., Zanão L.A.Z., Tomassoni F. (2013). The culture of pequi (*Caryocar brasiliense* Camb.). Acta Iguazu.

[5] Amaral L.F.B., Moriel P., Foglio M.A., Mazzola P.G. (2014). Evaluation of the Cytotoxicity and Phototoxicity of Caryocar Brasiliense Supercritical Carbon Dioxide Extract. BMC Complement. Altern. Med..

[6] Bezerra R.G. (2021). Pre-clinical evaluation of the pequi oil (*Caryocar coriaceum* Wittm.) and emulsion based on it for the treatment of dermatitis: Formulation, characterization and antimicrobial and anti-inflammatory effects. Master’s Dissertation.

[7] Viroli S.L.M., Rodrigues F.M., Viroli S.G., Carvalho N.P., Alves T.T., Lança A.C., Vivan J.V., Ramos M.L., Feitosa K.P., Veloso C. (2022). Extraction and Characterization of Oil from the Pulp of Pequi (*Caryocar* Brasiliense) Produced Manually in the Allotment Piauzinho Municipality of Pium-TO. Res. Soc. Dev..

[8] Araújo A.C.M.A., Menezes E.G.T., Terra A.W.C., Dias B.O., de Oliveira É.R., Queiroz F. (2018). Bioactive Compounds and Chemical Composition of Brazilian Cerrado Fruits’ Wastes: Pequi Almonds, Murici, and Sweet Passionfruit Seeds. Food Sci. Technol..

[9] Roll M.M., Miranda-Vilela A.L., Longo J.P.F., da Silveira Agostini-Costa T., Grisolia C.K. (2018). The Pequi Pulp Oil (*Caryocar brasiliense* Camb.) Provides Protection against Aging-Related Anemia, Inflammation and Oxidative Stress in Swiss Mice, Especially in Females. Genet. Mol. Biol..

[10] Cunha L.M.S., Pires R.F., dos Santos K.G., Dantas S.C. (2020). Comparison of yield by different methods of oil extraction from pequi pulp. Res. Soc. Dev..

[11] Pegorin Brasil G.S., Borges F.A., de Machado A.A., Mayer C.R.M., Udulutsch R.G., Herculano R.D., Funari C.S., dos Santos A.G., Santos L. (2022). A Sustainable Raw Material for Phytocosmetics: The Pulp Residue from the *Caryocar brasiliense* Oil Extraction. Rev. Bras. Farmacogn..

[12] Bonifácio B.V., da Silva P.B., Ramos M.A.D.S., Negri K.M.S., Bauab T.M., Chorilli M. (2014). Nanotechnology-Based Drug Delivery Systems and Herbal Medicines: A Review. Int. J. Nanomed..

[13] Lal H.M., Uthaman A., Thomas S., Lal H.M., Thomas S., Li T., Maria H.J. (2021). Silver Nanoparticle as an Effective Antiviral Agent. Polymer Nanocomposites Based on Silver Nanoparticles: Synthesis, Characterization and Applications.

[14] Uthaman A., Lal H.M., Thomas S., Lal H.M., Thomas S., Li T., Maria H.J. (2021). Fundamentals of Silver Nanoparticles and Their Toxicological Aspects. Polymer Nanocomposites Based on Silver Nanoparticles: Synthesis, Characterization and Applications.

[15] Barreto G.P.M., Benassi M.T., Mercadante A.Z. (2009). Bioactive Compounds from Several Tropical Fruits and Correlation by Multivariate Analysis to Free Radical Scavenger Activity. J. Braz. Chem. Soc..

[16] Tungmunnithum D., Thongboonyou A., Pholboon A., Yangsabai A. (2018). Flavonoids and Other Phenolic Compounds from Medicinal Plants for Pharmaceutical and Medical Aspects: An Overview. Medicines.

[17] Frasao B., Costa M., Silva F., Rodrigues B., Baltar J., Araujo J., Moreira D., Torrezan R., Conte-Junior C. (2018). Effect of Pequi (*Caryocar brasiliense*) and Juçara (*Euterpe Edulis*) Waste Extract on Oxidation Process Stability in Broiler Meat Treated by UV-C. PLoS ONE.

[18] Roesler R., Malta L.G., Carrasco L.C., Holanda R.B., Sousa C.A.S., Pastore G.M. (2007). Antioxidant activity of cerrado fruits. Food Sci. Technol..

[19] Magalhães F.S., de Souza Martins Sá M., Luiz Cardoso V., Hespanhol Miranda Reis M. (2019). Recovery of Phenolic Compounds from Pequi (*Caryocar brasiliense* Camb.) Fruit Extract by Membrane Filtrations: Comparison of Direct and Sequential Processes. J. Food Eng..

[20] Nascimento-Silva N.R.R., Mendes N.S.R., Silva F.A. (2020). Nutritional composition and total phenolic compounds content of pequi pulp (*Caryocar brasiliense* Cambess.). J. Bioenergy Food Sci..

[21] Ribeiro D.M., Fernandes D.C., Alves A.M., Naves M.M.V. (2014). Carotenoids Are Related to the Colour and Lipid Content of the Pequi (Caryocar Brasiliense Camb.) Pulp from the Brazilian Savanna. Food Sci. Technol..

[22] Malacrida C.R., Moraes I.C.F., de Rosso V.V., da Costa Rodrigues C.E., de Souza A.C. (2018). Effect of the Application of an Enzymatic Pretreatment on Bioactive Compounds of *Caryocar brasiliense* Camb Pulp Oil. J. Food Process. Preserv..

[23] Bakshi P.S., Selvakumar D., Kadirvelu K., Kumar N.S. (2020). Chitosan as an Environment Friendly Biomaterial—A Review on Recent Modifications and Applications. Int. J. Biol. Macromol..

[24] Coutinho A.J., Costa Lima S.A., Afonso C.M.M., Reis S. (2020). Mucoadhesive and PH Responsive Fucoidan-Chitosan Nanoparticles for the Oral Delivery of Methotrexate. Int. J. Biol. Macromol..

[25] De Oliveira T.D., Riani L.R., Costa M.P., Fabri R.L., Nascimento J.W.L., Silva F.P., da Silva Filho A.A., Tavares G.D. (2021). Baccharis Dracunculifolia Extract-Loaded Chitosan Nanoparticles: Development, Physicochemical Characterization and Cytotoxicity Evaluation. Braz. J. Dev..

[26] Mahmoudi R., Tajali Ardakani M., Hajipour Verdom B., Bagheri A., Mohammad-Beigi H., Aliakbari F., Salehpour Z., Alipour M., Afrouz S., Bardania H. (2019). Chitosan Nanoparticles Containing Physalis Alkekengi-L Extract: Preparation, Optimization and Their Antioxidant Activity. Bull. Mater. Sci..

[27] Mondéjar-López M., Rubio-Moraga A., López-Jimenez A.J., García Martínez J.C., Ahrazem O., Gómez-Gómez L., Niza E. (2022). Chitosan Nanoparticles Loaded with Garlic Essential Oil: A New Alternative to Tebuconazole as Seed Dressing Agent. Carbohydr. Polym..

[28] Abosabaa S.A., ElMeshad A.N., Arafa M.G. (2021). Chitosan Nanocarrier Entrapping Hydrophilic Drugs as Advanced Polymeric System for Dual Pharmaceutical and Cosmeceutical Application: A Comprehensive Analysis Using Box-Behnken Design. Polymers.

[29] Kaiser M., Pereira S., Pohl L., Ketelhut S., Kemper B., Gorzelanny C., Galla H.-J., Moerschbacher B.M., Goycoolea F.M. (2015). Chitosan Encapsulation Modulates the Effect of Capsaicin on the Tight Junctions of MDCK Cells. Sci. Rep..

[30] Doughty D. (1994). A Rational Approach to the Use of Topical Antiseptics. J. Wound Ostomy Cont. Nurs..

[31] Eming S.A., Wynn T.A., Martin P. (2017). Inflammation and Metabolism in Tissue Repair and Regeneration. Science.

[32] Wynn T.A., Vannella K.M. (2016). Macrophages in Tissue Repair, Regeneration, and Fibrosis. Immunity.

[33] Barman P.K., Koh T.J. (2020). Macrophage Dysregulation and Impaired Skin Wound Healing in Diabetes. Front. Cell Dev. Biol..

[34] Athira K., Keerthi T.R. (2016). Analyses of Methanol Extracts of Two Marine Sponges, Spongia Officinalis Var. Ceylonensis and Sigmadocia Carnosa from Southwest Coast of India for Their Bioactivities. Int. J. Curr. Microbiol. Appl. Sci..

[35] Kumar V., Bhat Z.A., Kumar D., Khan N., Chashoo I. (2012). Evaluation of Anti-Inflammatory Potential of Leaf Extracts of Skimmia Anquetilia. Asian Pac. J. Trop. Biomed..

[36] Dias G.T. (2018). Evaluation of In Silico Toxicity and In Vitro Hemolytic, Antioxidant and Antibacterial Activities of the Essential Oil and Microencapsulated Essential Oil forms of Lippia Pedunculosa.

[37] De Sousa A.P., Cordeiro L.V., da Silva Souza H.D., de Souza M.D.F.V., da Silveira R.D.C., de Oliveira Filho A.A. (2022). Evaluation of in vitro cytotoxicity and ex-vivo genotoxicity in compounds from Pavonia Glazioviana Gürke (Malvaceae). Rev. Ciências Médicas Biológicas.

[38] De Reis L.D.O., Nassif P.A.N., Tabushi F.I., Milléo F.Q., Favero G.M., Ariede B.L., Reis C.F.D.D., Dalabona B.F. (2016). Preliminary analysis of interleukin-6 changes in pre- and postoperative in diabetic patients with bmi < 35 submitted to partial duodenal switch. Arq. Bras. Cir. Dig..

[39] Grazia Roncarolo M., Gregori S., Battaglia M., Bacchetta R., Fleischhauer K., Levings M.K. (2006). Interleukin-10-Secreting Type 1 Regulatory T Cells in Rodents and Humans. Immunol. Rev..

[40] Iyer S.S., Cheng G. (2012). Role of Interleukin 10 Transcriptional Regulation in Inflammation and Autoimmune Disease. Crit. Rev. Immunol..

[41] De Torres O.L.R., de Santana F.C., Torres-Leal F.L., de Melo I.L.P., Yoshime L.T., Matos-Neto E.M., Seelaender M.C.L., Araújo C.M.M., Cogliati B., Mancini-Filho J. (2016). Pequi (*Caryocar brasiliense* Camb.) Almond Oil Attenuates Carbon Tetrachloride-Induced Acute Hepatic Injury in Rats: Antioxidant and Anti-Inflammatory Effects. Food Chem. Toxicol..

[42] Guarnier L.P., Romão P.V.M., Palozi R.A.C., Silva A.O., Lorençone B.R., Marques A.A.M., dos Santos A.C., Souza R.I.C., Souza K.D., Lourenço E.L.B. (2019). Development of a Predictive Model to Induce Atherogenesis and Hepato-Renal Impairment in Female Rats. Biomolecules.

[43] Di Santo M.C., D’ Antoni C.L., Domínguez Rubio A.P., Alaimo A., Pérez O.E. (2021). Chitosan-Tripolyphosphate Nanoparticles Designed to Encapsulate Polyphenolic Compounds for Biomedical and Pharmaceutical Applications—A Review. Biomed. Pharmacother..

[44] Darif D., Hammi I., Kihel A., El Idrissi Saik I., Guessous F., Akarid K. (2021). The Pro-Inflammatory Cytokines in COVID-19 Pathogenesis: What Goes Wrong?. Microb. Pathog..

[45] Diniz D.M. (2015). Anti-Inflammatory Activity of Microemulsion Containing Pequi Oil (Caryocar coriaceum W.).

[46] Santos E., de Araújo S.P., Moraes C.L.M., Araújo S.d.S., Raesel G., Justi P.N., Argandonac E.J.S., Kassuyac C. (2015). Antiedematogenic activity of Pequi *Caryocar brasiliense* oil. Exp. Clin. Perspect. Biomed. Innov. Health Educ. (PECIBES).

[47] Mansur M.C.P.P.R., Leitão S.G., Cerqueira-Coutinho C., Vermelho A.B., Silva R.S., Presgrave O.A.F., Leitão Á.A.C., Leitão G.G., Ricci-Júnior E., Santos E.P. (2016). In Vitro and In Vivo Evaluation of Efficacy and Safety of Photoprotective Formulations Containing Antioxidant Extracts. Rev. Bras. De Farmacogn..

[48] Santos L., Pegorin S.G., Machado A.A., Dos Santos A.G., Funari C.S., Ribeiro-Paes J.T. (2021). Method of Obtaining Phytocosmetic in Antioxidant and Photoprotective Gel Cream and Formulation of Phytocosmetic in Antioxidant and Photoprotective Gel Cream Containing Extract of Caryocar brasiliense Camb.-pequi.

[49] Serdar G., Sökmen M., Demir E., Sökmen A., Bektaş E. (2015). Extraction of Antioxidative Principles of Achillea Biserrata M. Bieb. and Chromatographic Analyses. Int. J. Second. Metab..

[50] Calvo P., Remuñan-López C., Vila-Jato J.L., Alonso M.J. (1997). Chitosan and Chitosan/Ethylene Oxide-Propylene Oxide Block Copolymer Nanoparticles as Novel Carriers for Proteins and Vaccines. Pharm. Res..

[51] Tsuboy M.S., Marcarini J.C., Luiz R.C., Barros I.B., Ferreira D.T., Ribeiro L.R., Mantovani M.S. (2010). In Vitro Evaluation of the Genotoxic Activity and Apoptosis Induction of the Extracts of Roots and Leaves from the Medicinal Plant *Coccoloba Mollis* (Polygonaceae). J. Med. Food.

[52] Azedo M.R., Blagitz M.G., Souza F.N., Benesi F.J., Della Libera A.M.M.P. (2011). Functional Evaluation of Monocytes in Cattle Naturally Infected with the Bovine Leucosis Virus. Arq. Bras. Med. Veterinária Zootec..

[53] Bastos C.R., Blagitz M.G., Souza F.N., Batista C.F., Stricagnolo C.R., Azedo M.R., Della Libera A.M.M.P. (2012). Cell viability, phagocytosis and spreading by mononuclear phagocytes and hydrogen peroxide release by leukocytes from healthy and infected bovine mammary glands. Pesqui. Vet. Bras..

[54] Ananthi T., Chitra M. (2013). Screening of in vitro anti-inflammatory activity of Michelia champaca linn. Asian J. Pharm. Clin. Res..

[55] Ethics Committee on the Use of Animals (2001). OECD/OCDE 423 OECD Guideline for Testing of Chemicals Acute Oral Toxicity-Acute Toxic Class Method Introduction.

[56] Winter C.A., Risley E.A., Nuss G.W. (1962). Carrageenin-Induced Edema in Hind Paw of the Rat as an Assay for Antiiflammatory Drugs. Proc. Soc. Exp. Biol. Med..

[57] Journal Officiel de la Republique Française (1996). Decree of December 27, 1996 Relating to the Methods of Analysis Necessary to Control the Composition of Cosmetic Products. Appendix IV: Official Method for Evaluating the Potential Irritant by Application to the Chorioallantoic Membrane of the Hen’s Egg.

